# Tailored Nutritional Intervention Based on AI-Assessed Dietary Assessment in Nutritionally Compromised Older Adults: Impact on Anthropometric, Cognitive, and EEG Parameters

**DOI:** 10.3390/nu18152415

**Published:** 2026-07-24

**Authors:** Yejin Seo, Soyoung Jung, Hae Jin Kang, Hee-Sook Lim, Ji Youn Hong, Jean Kyung Paik, Dayeon Shin, Yujung Lee, Seung Wan Kang, Yoo Kyoung Park

**Affiliations:** 1 Department of Medical Nutrition, Graduate School of East-West Medical Nutrition, Kyung Hee University, Yongin 17104, Republic of Korea; yejinalive@khu.ac.kr; 2 AgeTech-Service Convergence Major, Department of Medical Nutrition, Graduate School of East-West Medical Nutrition, Kyung Hee University, Yongin 17104, Republic of Korea; jsy0623@khu.ac.kr (S.J.); jjjjj@khu.ac.kr (H.J.K.); 3 AgeTech-Service Convergence Major, Department of Gerontology, Graduate School of East-West Medical Nutrition, Kyung Hee University, Yongin 17104, Republic of Korea; limhsgeron@khu.ac.kr; 4 Department of Food Regulatory Science, Korea University School, Sejong 30019, Republic of Korea; khjy1025@korea.ac.kr; 5 Department of Food and Nutrition, Eulji University, Seongnam 13135, Republic of Korea; jkpaik@eulji.ac.kr; 6 Department of Nutritional Science and Food Management, Ewha Womans University, Seoul 03760, Republic of Korea; dayeonshin@ewha.ac.kr; 7 Nuvilab, Inc., Seoul 06173, Republic of Korea; yujung.lee@nuvilab.com; 8 iMediSync, Inc., Seoul 06181, Republic of Korea; drdemian@snu.ac.kr; 9 National Standard Reference Data Center for Korean EEG, College of Nursing, Seoul National University, Seoul 03080, Republic of Korea

**Keywords:** artificial intelligence, nutrition assessment, malnutrition, cognitive dysfunction (mild cognitive impairment), dementia, assisted living facilities, residential facilities

## Abstract

**Objectives**: The aim of this study was to evaluate whether dietary intake-guided precision nutrition interventions, derived from AI-driven food intake monitoring, improve cognitive function and electroencephalographic (EEG) biomarkers in older adults receiving long-term care. **Methods**: A total of 108 adults aged ≥50 years were recruited from five long-term care facilities. The study included 4 weeks of AI-driven dietary data collection, 2 weeks of data analysis and participant grouping, and 4 weeks of targeted nutritional intervention. Dietary intake was assessed using an AI-based food scanner. Anthropometric measures, biochemical markers, nutritional and cognitive questionnaires, and EEG were evaluated at baseline and postintervention. Participants were classified into three groups based on nutrient intake: “severely inadequate,” “marginally inadequate,” and “adequate.” Tailored food-based interventions, including nut mixes, senior-friendly meat products, and oral nutritional supplements, were provided according to group classification. **Results**: Energy intake increased in all groups. Nutritional status and cognitive function improved primarily in the severely inadequate group, while favorable EEG changes were observed across all groups. Changes in dietary composition and lipid profiles were observed in the “adequate” group, whereas the “marginally inadequate” group showed no significant changes. **Conclusions**: AI-based monitoring and nutritional intervention may improve nutritional status, cognitive-related outcomes, and EEG biomarkers in older adults receiving long-term care, supporting the potential of precision nutrition approaches in this population. Clinical Trial Registration KCT0009558 (CRIS, Republic of Korea).

## 1. Introduction

Aging is a natural, irreversible process associated with physiological and functional changes that increase the risk of chronic disease, cognitive decline, and malnutrition in older adults. With a continuously increasing older adult population, geriatric syndromes requiring long-term care (LTC), such as dementia and severe stroke, are becoming increasingly prevalent. These demographic changes have increased interest in modifiable lifestyle factors, particularly dietary, as a strategy to delay age-related cognitive decline [1]. In Korea, the LTC insurance system was established to address geriatric health issues, and since its implementation, both LTC facilities and residents have steadily increased [2]. Aging-related physiological changes, including reduced sensory functions (sight, smell, and taste), impaired digestion and absorption, altered hormone secretion, and decreased chewing and swallowing ability, can limit dietary adequacy in older adults. In LTC settings, these limitations often require texture-modified meals, which are associated with reduced nutrient intake and lower meal quality compared with standard diets [3].

Cognitive decline is a major concern in LTC populations. According to the 2022 Long-Term Care Survey, a nationwide survey of long-term care services and facilities in Korea, 54.4% of residents have dementia, which increases the risk of malnutrition by impairing communication of needs (e.g., hunger) and limiting the ability to perform daily activities, such as handling eating utensils [2]. Growing evidence indicates that insufficient or imbalanced dietary intake may accelerate neurodegeneration and contribute to progression from mild cognitive impairment to dementia [4]. Depression, chronic illness, and socioeconomic disadvantages further exacerbate undernutrition in older adults, increasing the risk of frailty, falls, hospitalization, and reduced quality of life [2]. Given the close relationship between nutritional status and cognitive function, LTC facilities routinely use tools such as the mini-mental examination (MMSE) and cognitive impairment screening test to monitor cognitive status. However, these tools rely heavily on communication and literacy, underscoring the need for objective cognitive function biomarkers. Emerging technologies, including wearable devices and electroencephalography, are therefore being explored as objective methods for screening mild cognitive impairment (MCI) and dementia [4].

Regular dietary monitoring is also essential to prevent malnutrition and related health complications. However, traditional dietary assessment methods, such as 24 h recalls, food frequency questionnaires, and 3-day meal diaries, are often unsuitable for older adults with cognitive decline or limited literacy. Previous studies in LTC settings have estimated nutrient intake by measuring the amount of food left uneaten, but this method is time-consuming and dependent on trained dietitians [3]. Recently, AI-based food scanners have emerged as a more efficient tool for monitoring dietary intake and assessing eating patterns [5].

Despite the importance of providing nutritionally adequate meals for residents with age-related conditions, meal planning and food provision in LTC facilities are often managed by nonprofessional staff, such as cooks or social workers [6]. While nutrition education can support chronic disease management, its effectiveness is limited among residents with cognitive impairments or low educational attainment, highlighting the need for practical and direct nutritional interventions [4]. To date, tailored nutritional approaches for older adults in LTC settings remain limited [6]. AI-assisted dietary monitoring offers new opportunities to implement dietary intake-guided precision nutrition interventions targeting cognitive health in aging populations.

Therefore, this study aimed to evaluate the effectiveness of AI-supported, tailored nutritional interventions on health outcomes among older adults residing in care facilities with limited access to dietitians. Specifically, we examined whether dietary intake-guided precision nutrition improves cognitive function and EEG-based biomarkers in LTC residents.

## 2. Materials and Methods

### 2.1. Study Participants

The study was conducted in accordance with the Declaration of Helsinki and approved by the Institutional Review Board of Kyung Hee University (approval no. KHGIRB-23-222) on 8 June 2023. This investigator-initiated study, commissioned by the Ministry of Food and Drug Safety, invited community care facilities nationwide, including daycare centers and nursing homes, to participate. Eligible facilities were those without an in-house dietitian, that prepared meals on site, and had the infrastructure to support an AI-based food scanner for dietary assessment. The sample size was calculated based on a previous similar study [7]. Given that the previous study included 88 participants with an 18.5% attrition rate, 108 participants were recruited to achieve a target sample size of 88. Eligible participants were facility users who provided informed consent and consumed meals at the facility at least three times per week. Exclusion criteria included neurological disorders associated with cognitive decline, infectious or metabolic diseases that may affect cognitive function, psychiatric disorders, and inability to cooperate with the study. In total, five facilities were ultimately included, and 108 participants were enrolled.

### 2.2. Study Design

This 10-week study comprised 4 weeks of AI-driven data collection, 2 weeks of data analysis and participant clustering, and a subsequent 4-week nutritional intervention. A facility-based, non-randomized quasi-experimental design was used, as randomization was not feasible due to operational constraints in meal provision and care delivery. Researchers visited the participating care facilities at baseline (week 0) and postintervention (week 10) to conduct assessments, including anthropometric measurements, blood tests, electroencephalography (EEG), demographic surveys, and questionnaires administered by trained dietitians and researchers. During the first 4 weeks, caregivers used an AI-based food scanner (Nuvilab Co., Ltd., Seoul, Republic of Korea, https://www.nuvilab.com, accessed on 21 July 2026) to record food intake before and after participants’ lunch meals, three times per week. Nutrient intake data for 10 nutrients (carbohydrates, protein, fat, vitamin A, vitamin C, thiamine, riboflavin, calcium, iron, and sodium) were analyzed using hierarchical and K-means clustering. Based on these analyses, a final three-cluster solution was selected after consideration of cluster overlap and interpretability. Because the groups were derived using a data-driven clustering approach rather than predefined nutrient-specific cut-off values, no fixed cut-offs were applied. The final clusters were interpreted according to the overall nutrient intake patterns and labeled as adequate, marginally inadequate, and severely inadequate based on their relative nutritional profiles.

### 2.3. Dietary Intervention

Intervention foods were provided once daily, 5 days per week, for 4 weeks, in addition to participants’ regular meals and snacks. Interventions were tailored to each group’s nutritional risk profile and functional characteristics, including cognitive status, activities of daily living, and meal type (e.g., standard, minced, pureed diets). Participants received group-specific food-based interventions: a nut mix for the “adequate group,” a meat dish for the “marginally adequate” group, and oral nutritional supplements (ONSs, a nutritionally complete formula categorized as Foods for Special Medical Purposes) for the nutritionally “severely inadequate” group. The meat dish consisted of five rotating high-protein menus prepared with beef, chicken, or pork using senior-friendly foods. In Korea, senior-friendly foods are defined by the “Senior-Friendly Industry Promotion Act” as foods manufactured, processed, or cooked to support older adults with swallowing, chewing, or digestive difficulties [8]. The serving sizes and nutrient compositions of the foods provided are shown in Table 1. A thickening agent was also provided to participants with swallowing difficulties to facilitate intake.

### 2.4. Nutritional Assessments

#### 2.4.1. Anthropometric and Biochemical Parameters

Anthropometric and biochemical parameters were assessed at baseline (week 0) and after the 10-week intervention. Anthropometric measurements included height, weight, body mass index (BMI), body fat mass (BFM), percent body fat (PBF), skeletal muscle mass (SMM), systolic blood pressure (SBP), and diastolic blood pressure (DBP). Body composition was measured using the InBody 270 (Biospace Co., Ltd., Seoul, Republic of Korea) for daycare center participants, an analyzer designed for healthy adults, and the InBody S10 (Biospace Co., Ltd., Seoul, Republic of Korea) for non-ambulatory individuals. InBody S10 enables measurement in sitting or supine positions, making it suitable for nursing home residents. Standing height was measured using an ultrasound height measurement device (HuBDIC Co., Ltd., Anyang, Republic of Korea). For participants who were unable to stand, height was obtained from facility records or estimated using the half-arm span method. Biochemical parameters included hemoglobin and a lipid profile (total cholesterol, low-density lipoprotein (LDL) cholesterol, and high-density lipoprotein (HDL) cholesterol). All biochemical analyses were conducted using portable point-of-care testing devices using the Afinion Analyzer 2 (Abbott, Fornebu, Norway). Hemoglobin was also measured using the HemoCue Hb-301 (HemoCue AB, Angelholm, Sweden).

#### 2.4.2. Mini Nutritional Assessment-Short Form (MNA-SF)

To assess nutritional status, the MNA-SF was used at weeks 0 and 10. The MNA-SF consists of six questions derived from the original MNA and has been validated with high sensitivity, specificity, and correlation with the full MNA [9]. It classifies older individuals as well-nourished, at risk of malnutrition, or malnourished and serves as a valid, time-saving alternative. The MNA-SF includes dietary assessment (1 item), anthropometric measurement (1 item), and general physical and mental evaluations (4 items), with a total score of 14 points. Scores < 8 indicate malnutrition, scores between 8 and 11 indicate risk of malnutrition, and scores of ≥12 or indicate good nutritional status [10].

#### 2.4.3. Dietary Intake Assessment via AI Food Scanner

An AI food scanner (Nuvilab Co., Ltd., Seoul, Republic of Korea) was used to measure participants’ meal intake. Its accuracy was verified by the Korea Information and Communication Technology Association; it achieved over 95% accuracy in food classification and 92.9% accuracy in food volume estimation [5]. The device was installed along the dining area aisle, allowing caregivers to scan participants’ plates. Each participant was assigned an ID card, which caregivers used to tag the scanner before and after each meal. Meal intake was automatically calculated by estimating portion size and subtracting leftovers. Data, including identified menu items and food volume, were transmitted to a server for analysis. Nutrient intake was estimated using the Food and Nutrition Composition Database of the Korea Ministry of Food and Drug Safety stored on the server. The analyzed data provided individualized information on nutrient intake, nutrient composition, time to meal completion, and particular eating habits. This dietary intake assessment facilitated efficient collection of participants’ dietary intake-related information and to better understand their eating behaviors.

### 2.5. Neurocognitive Assessments

#### 2.5.1. Korean Version of the Mini-Mental State Examination-2: Standard Version

Cognitive function was evaluated using the Korean version of the Mini-Mental State Examination-2: Standard Version (K-MMSE-2:SV; Blue Form) [11]. This brief test screens for cognitive impairment, assesses severity, and monitors cognitive changes over time [12]. The K-MMSE-2:SV evaluates seven cognitive domains: registration (3 points), orientation to time (5 points), orientation to place (5 points), recall (3 points), attention and calculation (5 points), language (8 points), and drawing (1 point). The total score ranges from 0 to 30 points and is calculated by summing all item scores [12].

#### 2.5.2. EEG

EEG was used to assess cognitive impairment using the iSyncWave (iMediSync, Seoul, Republic of Korea, http://www.imedisync.com, accessed on 21 July 2026), a dry wireless helmet with built-in transcranial photobiomodulation (tPBM). The device connects to the company’s mobile application, iSyncMe (iMediSync, Seoul, Republic of Korea, http://www.imedisync.com, accessed on 21 July 2026), and transmits data to the cloud-based platform iSyncBrain (iMediSync, Seoul, Republic of Korea, http://www.imedisync.com, accessed on 21 July 2026), for AI-driven quantitative electroencephalography (QEEG) analysis. Measurements were performed for 2 min and 30 s under resting-state conditions with the eyes open and closed. Participants were instructed to remain still, relaxed, and awake during the assessment.

### 2.6. Statistical Analysis

Data were analyzed using the Statistical Package for the Social Sciences version 28.0 (IBM Corp., Armonk, NY, USA). Data normality was assessed using the Shapiro–Wilk test at a 5% significance level. Continuous variables are presented as means and standard deviations, and categorical variables as frequencies and percentages. The chi-square test or Fisher’s exact test was used for categorical variables. Changes between baseline and after the 10-week intervention were analyzed using paired *t*-tests or the Wilcoxon signed-rank test. Statistical significance was defined as a *p*-value of 0.05.

## 3. Results

### 3.1. Flow Chart of Participants, General Characteristics, and Health Status of Participants

**Figure 1:**
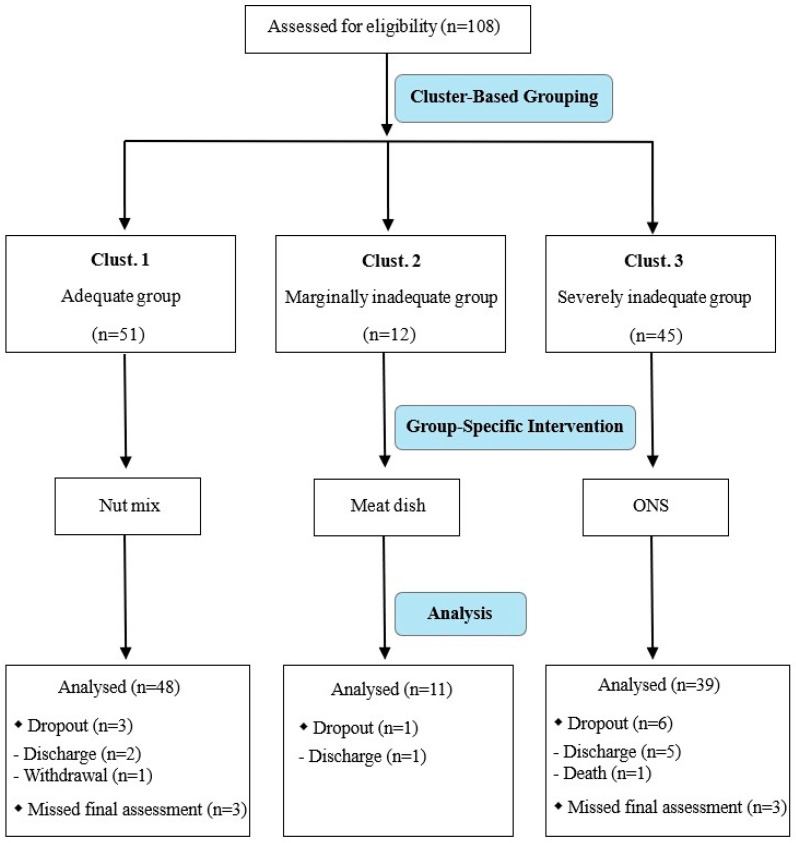
Flow chart of participants. A total of 108 participants were classified into three nutritional intake-based clusters: adequate (*n* = 51), marginally inadequate (*n* = 12), and severely inadequate (*n* = 45). Each group received a 10-week tailored intervention: nut mix, senior-friendly foods, or oral nutritional supplements, respectively. Final analyses included 48, 11, and 39 participants after accounting for dropouts.

A total of 108 participants were enrolled, and 98 participants were included in analysis, yielding a 9.2% attrition rate (Figure 1). Participants were clustered into three groups based on nutrient intake during the first 4 weeks: “adequate” (*n* = 51; 13 males and 38 females), “marginally inadequate” (*n* = 12; 2 males and 10 females), and “severely inadequate” (*n* = 45; 11 males and 34 females). Mean ages were 85.2 ± 7.22, 80.6 ± 11.30, and 82.6 ± 9.20 years, respectively, with no significant differences (*p* = 0.246). More than 75.0% of participants had a middle school education or lower. The mean number of comorbidities was 3.05 ± 1.16, with no significant differences among groups (*p* = 0.129). Medical histories (including MCI dementia, diabetes, hypertension, and cardiovascular disease), hearing status, and number of residual teeth were also comparable across groups.

### 3.2. Anthropometric and Biochemical Parameter Changes

Within-group changes in anthropometric and biochemical parameters after the 10-week intervention are summarized in Table 2. In the “adequate” group, significant increases were observed in body weight, BFM, PBF, midarm circumference, and calf circumference. For lipid profiles, low-density lipoprotein cholesterol decreased, whereas high-density lipoprotein cholesterol and hemoglobin levels increased. Blood pressure parameters showed a significant increase postintervention. In the “severely inadequate” group, BFM, PBF, total cholesterol, and high-density lipoprotein cholesterol increased postintervention. In contrast, no significant within-group changes were observed in the “marginally inadequate” group after the 10-week intervention.

### 3.3. Nutrient Intake and Nutrition Status Changes

**Figure 2:**
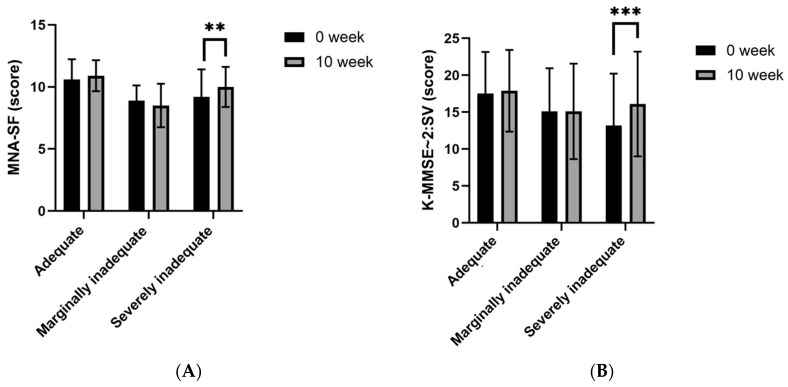
Changes in the MNA-SF and K-MMSE~2:SV scores of the study participants after the 10-week intervention. (**A**) MNA-SF scores and (**B**) K-MMSE-2:SV scores. Values are the mean ± SD. Paired *t*-tests or Wilcoxon signed-rank tests were used. Significance levels: ** *p* < 0.01, *** *p* < 0.001.

Dietary intake changes at pre- and postintervention are summarized in Table 3. The total energy intake increased across all three groups following the intervention. In the “adequate” intake group, carbohydrate and fat intake increased, along with higher intakes of unsaturated fatty acids and essential amino acids. The “marginally inadequate” group showed increased protein and essential amino acid intake, whereas the “severely inadequate” group demonstrated significant increases across all macronutrients. For micronutrients, thiamine, vitamin B_6_, and vitamin E intake increased across all groups. Additional improvements in selected vitamins and minerals were observed depending on baseline nutritional status, with broader increases in the “severely inadequate” group. Overall, mineral intake increased across groups, with reduced sodium intake observed in the adequate intake group. Average changes in MNA-SF after the 4-week intervention are shown in Figure 2A. Average changes in MNA-SF scores were observed in the “severely inadequate group,” whereas no significant changes were observed in the “adequate group” or “marginally inadequate group” (Table 4).

### 3.4. K-MMSE-2:SV Score Changes

The average changes in K-MMSE-2:SV scores postintervention are shown in Figure 2B. No significant within-group changes were observed in the “adequate” or “marginally inadequate” groups, whereas the “severely inadequate” group showed significant improvements in both nutritional status and cognitive function. Detailed changes in K-MMSE-2:SV subdomains are presented in Table 5.

### 3.5. EEG Changes

Changes in EEG frequency across five brain regions after the 10-week intervention are summarized in Table 6. Reductions in delta power, associated with improved cognitive function, were observed in the frontal lobe across all groups, with additional decreases in the central sulcus except in the “severely inadequate” group. Increases in alpha-2 power were observed in the central sulcus, temporal, and parietal lobes of the adequate intake group, whereas no significant changes were seen in the “marginally inadequate” group. The severely deficient group showed increases in alpha-2 power in the parietal lobe. Similarly, beta-1 power increased in the frontal lobe, central sulcus, and parietal lobe regions of the “adequate” intake group, whereas no significant changes were observed in the “marginally inadequate” group.

## 4. Discussion

Using AI-driven dietary assessment and tailored interventions, this study addresses challenges in nutritional management where individualized approaches are difficult to implement. Participants were clustered by nutrient intake patterns and subsequently received 4-week tailored interventions based on nutritional status, meal type, cognitive function (K-MMSE-2:SV), and functional status (K-ADL). To overcome communication barriers in facility settings, health data were collected using AI-based food scanners and EEG devices. Supplementary foods were then provided based on these assessments, resulting in observed improvements in nutrient intake and EEG indicators across all groups.

Distinct changes in dietary intake and nutritional indicators were observed across all groups. In the “adequate” group, nut supplementation was associated with increased energy, carbohydrate, and fat intake. Cholesterol intake decreased, while monounsaturated fatty acid (MUFA) intake increased approximately fourfold. Improvements in blood lipid profiles were also observed, with increased HDL cholesterol and decreased LDL cholesterol, likely reflecting the high content of MUFAs and PUFAs, phytosterols, and antioxidants in nuts, consistent with previous findings [13,14]. Intake of B vitamins, vitamin E, and minerals such as magnesium, selenium, and zinc was also increased. Although hematopoietic nutrients such as iron and vitamin B_12_ did not increase, hemoglobin levels improved, possibly due to reduced oxidative stress from micronutrient supplementation in nuts [13,15]. Furthermore, calf circumference, a key predictor of mortality in older adults, increased [16].

In the “marginally inadequate” group, consumption of senior-friendly meat dishes may have contributed to increased energy, protein, and vitamin B_1_ intake. However, changes in meal composition appeared to reduce vitamin A and C intake. Unlike the other groups, no statistically significant improvements were observed in nutritional status indicators, including MNA-SF scores, blood lipid profiles, or anemia. This may be due to the small sample size (*n* = 11) and participant heterogeneity, including differences in medical history.

In the “severely inadequate” group, MNA-SF and K-MMSE-2:SV scores significantly increased, indicating improvements in nutritional status and cognitive function. Caloric intake increased by 66% and protein intake by 70%. Body weight increased, while skeletal muscle mass (SMM) remained stable. In contrast, BFM and PBF showed modest increases, which may reflect a physiological restoration in older adults, as body composition changes during malnutrition are more pronounced, and muscle mass recovery is often limited with aging [17]. Although cholesterol levels increased, they remained within the normal range (≤200 mg/dL), which has been associated with improved survival outcomes in bedridden individuals [18].

Regarding cognitive function and EEG findings, the EEG showed significant changes in brainwave patterns across all groups, suggesting potential neurophysiological improvements associated with cognitive function. Reduced delta activity, a cognitive decline marker, were observed in all groups. K-MMSE-2:SV scores significantly improved in the “severely inadequate” group, with EEG findings suggesting increased neural and possible compensatory activity in specific brain regions. In the “adequate” group, increased alpha-2, delta, and beta-1 power were observed in the parietal lobe; these are patterns that have previously been reported in studies of cognitive decline and early Alzheimer’s disease [19]. These changes may reflect alterations in neural activity and potential stabilization of cognitive decline. Improvements in brainwave activity may partly reflect mastication during nut consumption as well as the neuroprotective effects of nutrients contained in nuts. Nuts are rich in monounsaturated and poly-unsaturated fatty acids, vitamin E, magnesium, and zinc, which may reduce oxidative stress, stabilize neuronal membranes, and support synaptic plasticity [14,20]. Furthermore, chewing ability in older adults has been associated with overall health, including effects on heart rate and blood circulation, which may have contributed to these cognitive benefits [21].

In the “marginally inadequate” group, reduced frontal delta activity suggested partial attenuation of dementia-related EEG patterns [21].

In the “severely inadequate” group, reductions in delta waves, increased frontal beta power, and increased parietal alpha-2 power were observed. These changes may reflect cortical activity related to attention, calculation, and language, consistent with improvements in specific K-MMSE-2:SV subcategories [22,23,24].

While K-MMSE-2:SV scores showed limited sensitivity to early cognitive changes, EEG provided objective neurophysiological data that complemented conventional cognitive assessments. These EEG findings are consistent with previous studies showing that reductions in delta activity and increases in alpha activity are associated with improved cognitive function in older adults with MCI and dementia [25]. In the present study, the concurrent improvements in EEG activity and K-MMSE-2:SV scores further support the complementary value of EEG as an objective measure of cognitive function. Therefore, the combined use of EEG and conventional cognitive tests may be useful for evaluating cognitive changes in older adults with MCI or early-stage dementia [26]. Overall, the improvement in cognitive function observed in the severely nutritionally inadequate group may be clinically meaningful because it occurred alongside improvements in nutritional status and EEG parameters. Although regression to the mean cannot be completely excluded because of the poorer baseline nutritional and cognitive status of this group, the concurrent improvements across multiple outcomes suggest that the findings are unlikely to be explained solely by regression to the mean.

This study has several limitations. First, the absence of a control group limited direct comparison of intervention effects. Although a randomized controlled design would have provided stronger evidence, implementing such a design was not feasible because providing the intervention to only some residents within the same long-term care facility raised ethical and practical concerns and was likely to result in contamination between participants. In addition, using a separate facility as a control could have introduced substantial bias due to heterogeneity in resident characteristics, baseline nutritional status, staffing, and meal-service operations across facilities. Given these constraints, this study employed a non-randomized quasi-experimental design, causal relationships between the intervention and the observed outcomes cannot be established. Although the intervention was implemented within routine care by existing facility staff, alternative explanations for the observed improvements, including increased attention from care staff during the intervention period and temporal changes, cannot be completely excluded. Therefore, the findings should be interpreted with caution, and future studies including appropriate comparison groups are warranted to confirm these findings.

Second, individual adherence to the intervention foods was not directly assessed. Consequently, the actual amount of intervention foods consumed by each participant could not be confirmed. Although overall meal consumption was monitored using AI-based tray scanning, snack intake and food consumed outside the monitored meals were not comprehensively assessed, which may have affected estimates of total nutrient intake.

Third, monitoring errors may have occurred due to inconsistent menu documentation in facilities without dietitians. In these facilities, the lack of detailed cooking instructions and direct communication with cooks made it difficult to identify exact ingredients and preparation methods, which may have affected the accuracy of nutrient estimation.

In addition, because participants received different intervention foods according to their nutritional profiles, it was not possible to distinguish the independent effects of the personalized nutritional stratification approach from those of the intervention foods.

Finally, participants were recruited from selected long-term care facilities; therefore, caution is warranted when generalizing these findings to the broader Korean older adult population.

Despite these limitations, this study demonstrates the feasibility of integrating AI-basted dietary assessment, nutritional stratification, and tailored interventions in LTC facilities. These findings provide preliminary evidence to support future controlled studies evaluating AI-assisted nutritional management in older adults.

## 5. Conclusions

This study demonstrates the feasibility of implementing tailored nutritional interventions using AI-driven digital health data to improve nutritional and cognitive outcomes in older adults in care facilities. This group-based approach has the potential to support nutritional and cognitive health as the LTC population grows. Notably, changes in cognitive function and EEG biomarkers provide preliminary evidence that dietary intake-based precision nutrition may be associated with cognitive-related outcomes and cognitive health in aging populations. Further research, including studies with appropriate comparison groups, is needed to optimize and expand its application in real-world settings.

## Figures and Tables

**Table 1:** Nutrient contents of foods provided during the intervention in three groups.

Nutrients	Adequate	Marginally Inadequate					Severely Inadequate
	Nut Mix ^(1)^	Main Side Dish A ^(2)^	Main Side Dish B ^(3)^	Main Side Dish C ^(4)^	Main Side Dish D ^(5)^	Main Side Dish E ^(6)^	ONS ^(7)^
Serving size	20 g	100 g	100 g	100 g	100 g	100 g	200 mL
Energy (kcal)	105.0	86.4	112.5	156.0	83.4	81.0	200.0
Macronutrients							
Protein (g)	3.0	10.8	14.5	11.2	10.7	8.8	8.0
Fat (g)	6.0	1.1	2.2	7.7	0.4	9.6	7.0
Carbohydrate (g)	10.0	8.3	8.5	10.0	9.2	16.2	27.0
MUFA (g)	4.9	1.8	1.9	4.4	2.4	0.9	0.0
PUFA (g)	4.1	0.5	0.1	0.9	2.7	0.0	0.0
Saturated fat (g)	0.7	0.5	1.0	3.5	0.2	2.2	2.0
Cholesterol (mg)	0.0	32.0	35.0	121.3	35.6	18.8	0.0
Vitamins							
Thiamine (mg)	0.1	0.3	0.1	0.2	0.5	0.1	0.4
Riboflavin (mg)	0.1	0.2	0.2	0.2	0.1	0.2	0.4
Niacin (NE) (mg)	0.5	3.2	3.7	3.8	6.4	0.2	2.0
Vit B_6_ (mg)	0.0	0.0	0.0	0.0	0.0	0.0	0.5
Folic acid (µg)	7.6	10.3	12.1	14.2	10.6	9.0	60.0
Vit B_12_ (µg)	0.0	0.4	0.8	0.5	0.3	0.0	1.0
Vit C (mg)	0.0	1.2	1.3	5.3	2.3	1.3	30.0
Vit A (μg RAE)	0.5	6.3	4.0	24.2	21.9	43.7	200.0
Vit E (mg α-TE)	0.7	0.2	0.1	0.8	0.5	0.0	6.0
Minerals							
Calcium (mg)	25.3	12.8	10.9	37.6	14.3	9.0	160.0
Iron (mg)	0.7	0.5	1.8	1.2	0.8	0.6	2.4
Zinc (mg)	0.6	1.4	3.5	1.8	1.5	0.6	2.2
Selenium (μg)	1.5	11.8	15.2	13.2	14.3	0.3	10.0

^(1)^ Nut mix: contains walnuts, almonds, cashews, macadamias, and dried blueberries. ^(2)^ A: Braised pork in soy sauce prepared using a “saturated steam technique” that controls pressure and temperature for a tender texture; includes okra, garlic, and onions. ^(3)^ B: Braised beef in soy sauce—prepared using a “saturated steam technique” that controls pressure and temperature for a tender texture; includes okra, garlic, and onions. ^(4)^ C: Hamburger steak—made using a tenderization technique, this dish is a mix of pork and beef in tomato sauce with cheese, mushrooms, and onions. ^(5)^ D: Stir-fried pork was prepared using tenderization techniques, which help maintain a consistently soft texture, facilitating easier digestion. ^(6)^ E: Boneless braised chicken made using a “saturated steam technique,” which controls pressure and temperature to achieve a tender texture and includes potatoes, carrots, and onions. ^(7)^ ONSs: oral nutritional supplements, standard-formulated food (drink) for special medical purposes.

**Table 2:** Changes in anthropometric and biochemical parameters of study participants after the 10-week intervention.

	Adequate ^(1)^ (*n* = 41)			Marginally Inadequate ^(2)^ (*n* = 11)			Severely Inadequate ^(3)^ (*n* = 35)		
	0 Week	10 Weeks	*p*-Value ^(4)^	0 Week	10 Weeks	*p*-Value ^(4)^	0 Week	10 Weeks	*p*-Value ^(4)^
Height (cm)	153.1 ± 8.6	153.1 ± 8.6	1.000	152.3 ± 12.0	152.3 ± 12.0	1.000	155.6 ± 7.7	155.6 ± 7.7	1.000
Weight (kg)	55.1 ± 9.6	55.6 ± 9.6	0.048 *	57.9 ± 12.6	57.5 ± 12.9	1.000	57.7 ± 9.5	58.0 ± 9.3	0.106
BMI (kg/m^2^)	23.5 ± 3.5	23.7 ± 3.5	0.080	24.8 ± 3.0	24.6 ± 2.9	1.000 ^(5)^	23.9 ± 3.9	24.0 ± 3.8	0.111
SMM ^(6)^ (kg)	19.4 ± 3.5	19.5 ± 4.8	0.860	17.8 ± 6.2	17.4 ± 6.1	0.379	18.4 ± 4.1	18.0 ± 4.3	0.060
BFM ^(7)^ (kg)	17.8 ± 6.5	18.9 ± 6.6	0.001 **	23.4 ± 7.1	23.9 ± 5.7	0.526	22.1 ± 6.6	23.6 ± 6.6	0.000 ***
PBF ^(8)^ (%)	31.7 ± 8.3	33.3 ± 7.8	0.008 **	40.6 ± 10.7	41.9 ± 8.1	0.287	38.5 ± 8.4	40.5 ± 9.0	0.000 ***
MAC ^(9)^ (cm)	25.2 ± 2.4	25.9 ± 3.0	0.006 **	25.2 ± 3.2	25.9 ± 2.6	0.260	25.4 ± 4.3	25.1 ± 3.4	0.409
CC ^(10)^ (cm)	31.6 ± 3.5	32.3 ± 3.1	0.000 ***	29.4 ± 4.3	29.9 ± 3.4	0.688 ^(5)^	30.3 ± 5.1	29.9 ± 5.5	0.080
SBP ^(11)^ (mmHg)	121.7 ± 20.9	127.7 ± 18.5	0.055	133.3 ± 16.5	121.6 ± 17.9	0.156	116.9 ± 17.2	129.2 ± 16.8	0.000 ***
DBP ^(12)^ (mmHg)	65.9 ± 10.8	69.3 ± 9.9	0.036 *	75.4 ± 9.9	73.0 ± 14.1	0.608	68.3 ± 11.7	75.8 ± 11.1	0.000 ***
Total chol (mg/dL)	159.0 ± 33.9	160.9 ± 31.3	0.554	159.6 ± 41.5	175.6 ± 47.1	0.055	152.2 ± 31.4	165.7 ± 36.9	0.000 ***
LDL ^(13)^ chol (mg/dL)	74.5 ± 25.6	65.7 ± 29.3	0.004 *	76.5 ± 32.5	88.7 ± 36.8	0.066 ^(5)^	67.4 ± 26.4	70.9 ± 30.8	0.265
HDL ^(14)^ chol (mg/dL)	52.6 ± 14.1	60.5 ± 14.9	0.000 ***	44.1 ± 9.8	48.8 ± 10.5	0.062	47.3 ± 14.1	55.8 ± 13.9	0.000 ***
Hb (g/dL)	11.6 ± 1.7	12.1 ± 1.5	0.017 *	11.7 ± 2.8	11.5 ± 2.6	0.663	11.3 ± 1.5	11.6 ± 1.7	0.263

Values are presented as the mean ± SD. ^(1)^ Adequate: Participants were clustered as an adequate intake group and provided with nut mix. ^(2)^ Partial deficient: Participants were clustered as the partial deficient intake group and provided with meat dishes (senior-friendly foods). ^(3)^ Total deficient: Participants were clustered as the total deficient intake group and provided with ONSs. ^(4)^ Significance was determined by a paired *t*-test with a *p*-value less than *p* < 0.05. * *p* < 0.05, ** *p* < 0.01, *** *p* < 0.001. ^(5)^ Significance was determined using the Wilcoxon signed-rank test, with a *p*-value less than *p* < 0.05. ^(6)^ SMM—skeletal muscle mass. ^(7)^ BFM—body fat mass. ^(8)^ PBF—percent body fat. ^(9)^ MAC—midarm circumference. ^(10)^ CC—calf circumference. ^(11)^ SBP—systolic blood pressure. ^(12)^ DBP—diastolic blood pressure. ^(13)^ LDL—low-density lipoprotein. ^(14)^ HDL—high-density lipoprotein.

**Table 3:** Nutrient intake of lunch meal at 0 and 10 weeks (data for 10 weeks include the nutrients derived from the intervention; nut mix, meat dish and ONS).

Nutrients	Adequate (*n* = 45)			Marginally Inadequate (*n* = 10)			Severely Inadequate (*n* = 37)		
	0 Week	10 Weeks	*p*-Value ^(1)^	0 Week	10 Weeks	*p*-Value ^(1)^	0 Week	10 Weeks	*p*-Value ^(1)^
Energy (kcal)	430.3 ± 75.9	564.1 ± 93.0	0.000 ***	425.5 ± 90.3	524.0 ± 106.1	0.010 ***	349.2 ± 114.1	580.7 ± 140.6	0.000 ***
Carbohydrates (g)	65.5 ± 9.6	73.0 ± 13.1	0.000 ***	65.1 ± 13.6	79.1 ± 12.7	0.647	52.2 ± 19.5	80.0 ± 20.7	0.000 ***
Protein (g)	18.4 ± 4.7	19.3 ± 4.4	0.131	16.8 ± 4.2	25.5 ± 7.4	0.001 ***	14.0 ± 4.8	23.7 ± 6.3	0.000 ***
Fat (g)	9.7 ± 3.2	21.7 ± 3.8	0.000 ***	10.2 ± 2.9	11.0 ± 3.1	0.441	8.9 ± 2.9	18.3 ± 4.6	0.000 ***
C:P:F (%)	61.4:17.0:20.0	51.7:13.6:34.8	-	61.4:15.8:21.3	61.1:19.2:18.7	-	58.5:16.4:24.0	54.8:16.3:28.9	-
Cholesterol (mg)	33.3 ± 15.2	17.5 ± 6.9	0.000 ***	10.5 ± 3.7	36.7 ± 13.4	0.002 **	34.6 ± 19.1	37.9 ± 23.0	0.374
MUFA ^(3)^ (g)	2.1 ± 0.8	8.2 ± 1.0	0.000 ***	2.4 ± 0.7	2.6 ± 0.9	0.548	2.5 ± 0.9	3.1 ± 1.1	0.000 ***
PUFA ^(4)^ (g)	2.2 ± 1.0	8.3 ± 1.3	0.000 ***	4.0 ± 1.1	4.9 ± 1.3	0.070	3.5 ± 1.1	3.4 ± 1.0	0.887
Thiamine (mg)	0.2 ± 0.09	0.3 ± 0.1	0.001 **	0.2 ± 0.1	0.3 ± 0.1	0.042 *	0.2 ± 0.1	0.6 ± 0.1	0.000 ***
Riboflavin (mg)	0.3 ± 0.1	0.3 ± 0.1	0.000 ***	0.3 ± 0.1	0.2 ± 0.09	0.047 * ^(2)^	0.2 ± 0.1	0.6 ± 0.1	0.000 ***
Niacin (NE) (mg)	1.6 ± 0.6	2.4 ± 0.7	0.000 ***	1.7 ± 0.5	3.0 ± 1.4	0.209	2.0 ± 0.6	4.4 ± 0.8	0.000 ***
Vit B_6_ (mg)	0.1 ± 0.04	0.1 ± 0.1	0.000 ***	0.03 ± 0.01	0.04 ± 0.01	0.022 *	0.06 ± 0.03	0.6 ± 0.1	0.000 ***
Folic acid (µg)	77.8 ± 42.6	72.2 ± 22.3	0.314	44.8 ± 12.2	54.0 ± 19.1	0.141	58.1 ± 18.7	129.7 ± 31.6	0.000 ***
Vit B_12_ (µg)	0.8 ± 0.1	0.2 ± 0.1	0.000 ***	0.3 ± 0.1	0.3 ± 0.1	0.429	0.4 ± 0.1	1.2 ± 0.1	0.000 ***
Vit C (mg)	13.6 ± 7.3	10.3 ± 4.8	0.001 **	19.4 ± 4.8	14.3 ± 4.1	0.016 *	7.8 ± 2.8	41.5 ± 6.2	0.000 ***
Vit A (μg RAE)	120.7 ± 97.1	116.3 ± 50.6	0.710	83.2 ± 18.1	48.2 ± 15.4	0.000 ***	59.1 ± 23.7	296.3 ± 55.8	0.000 ***
Vit E (mg α-TE)	0.9 ± 0.5	1.8 ± 0.3	0.000 ***	1.5 ± 0.3	1.4 ± 0.4	0.724	0.9 ± 0.3	6.9 ± 0.3	0.000 ***
Calcium (mg)	89.0 ± 42.7	129.4 ± 34.4	0.000 ***	131.2 ± 28.5	130.2 ± 14.1	0.924	96.9 ± 32.1	260.8 ± 46.3	0.000 ***
Magnesium (mg)	61.6 ± 26.3	98.5 ± 21.6	0.000 ***	63.6 ± 17.2	70.9 ± 25.4	0.246	57.5 ± 18.4	124.2 ± 22.0	0.000 ***
Iron (mg)	2.8 ± 0.9	3.0 ± 0.7	0.215	3.0 ± 0.6	4.3 ± 1.6	0.007 **	2.2 ± 0.8	4.8 ± 1.1	0.000 ***
Zinc (mg)	2.0 ± 0.6	2.4 ± 0.5	0.001 **	1.3 ± 0.4	1.5 ± 0.5	0.209	1.5 ± 0.4	4.2 ± 1.0	0.000 ***
Selenium (μg)	15.3 ± 4.8	16.4 ± 4.3	0.048 *	11.8 ± 4.3	12.1 ± 5.4	0.877	13.5 ± 4.1	23.2 ± 4.4	0.000 ***

Values are presented as the mean ± SD. ^(1)^ Significance as determined by a paired *t*-test with a *p*-value less than *p* < 0.05. ^(2)^ Significance as determined by the Wilcoxon signed-rank test with a *p*-value less than *p* < 0.05. ^(3)^ MUFA—monounsaturated fatty acids. ^(4)^ PUFA—polyunsaturated fatty acids. * *p* < 0.05, ** *p* < 0.01, *** *p* < 0.001.

**Table 4:** Changes in the MNA-SF score of participants after the 10-week intervention.

	Adequate (*n* = 45)			Marginally Inadequate (*n* = 11)			Severely Inadequate (*n* = 37)		
	0 Week	10 Weeks	*p*-Value ^(1)^	0 Week	10 Weeks	*p*-Value ^(1)^	0 Week	10 Weeks	*p*-Value ^(1)^
MNA-SF ^(2)^ score	10.6 ± 1.63	10.9 ± 1.25	0.137	8.9 ± 1.22	8.5 ± 1.75	0.211	9.2 ± 2.22	10.0 ± 1.62	0.003 **
Well nourished	19 (42.2)	20 (44.4)		0 (0.0)	1 (9.1)	-	5 (13.5)	8 (21.6)	
At risk of malnutrition	25 (55.6)	25 (55.6)		9 (81.8)	6 (54.5)	-	24 (64.9)	27 (73.0)	
Malnourished	1 (2.2)	0 (0.0)		2 (18.2)	4 (36.4)	-	8 (21.6)	2 (5.4)	

Values are presented as means ± standard deviations. ^(1)^ Significance as determined by a paired *t*-test, with a *p*-value of <0.05. ^(2)^ MNA-SF—Mini Nutrition Assessment Tool. ** *p* < 0.01.

**Table 5:** Changes in K-MMSE~2 scores of participants after the 10-week intervention.

	Adequate (*n* = 45)			Marginally Inadequate (*n* = 11)			Severely Inadequate (*n* = 37)		
	0 Week	10 Weeks	*p*-Value ^(1)^	0 Week	10 Weeks	*p*-Value ^(2)^	0 Week	10 Weeks	*p*-Value ^(1)^
K-MMSE~2 ^(3)^ score	17.5 ± 5.65	17.9 ± 5.53	0.553	15.1 ± 5.84	15.1 ± 6.47	1.000	13.2 ± 7.02	16.1 ± 7.10	0.000 ***
Memory registration	2.6 ± 0.77	2.6 ± 0.92	0.618	2.1 ± 1.14	2.5 ± 1.04	0.339	2.3 ± 1.04	2.5 ± 0.93	0.073
Orientation to time	1.7 ± 1.75	1.8 ± 1.59	0.776	1.8 ± 1.33	1.9 ± 1.58	1.000	1.4 ± 1.54	1.6 ± 1.56	0.405
Orientation to the place	3.3 ± 1.56	3.4 ± 1.42	0.800	2.5 ± 1.37	2.6 ± 1.43	0.958	2.0 ± 1.56	2.4 ± 1.54	0.134
Memory recall	0.6 ± 0.84	0.6 ± 0.96	0.736	1.3 ± 1.19	1.0 ± 1.26	0.317	0.6 ± 1.04	0.7 ± 1.15	0.360
Attention and calculation	2.2 ± 1.89	2.3 ± 1.79	0.448	0.8 ± 1.40	0.8 ± 1.47	1.000	1.0 ± 1.38	1.8 ± 1.95	0.002 **
Language	6.6 ± 1.66	6.8 ± 1.51	0.550	6.1 ± 1.92	5.9 ± 2.21	0.680	5.3 ± 2.55	6.6 ± 1.89	0.000 ***
Drawing	0.5 ± 0.50	0.5 ± 0.51	0.643	0.5 ± 0.52	0.4 ± 0.50	0.564	0.4 ± 0.49	0.3 ± 0.45	0.103

Values are presented as means ± standard deviations or numbers (%). ^(1)^ Significance as determined by a paired *t*-test with a *p*-value < 0.05. ^(2)^ Significance as determined by the Wilcoxon signed-rank test with a *p*-value < 0.05. ^(3)^ K-MMSE~2—Korean version of the mini-mental state examination. ** *p* < 0.01, and *** *p* < 0.001.

**Table 6:** EEG changes in the study participants after the 10-week intervention.

	Adequate (*n* = 43)			Marginally Inadequate (*n* = 11)			Severely Inadequate (*n* = 36)		
	0 Week	10 Weeks	*p*-Value ^(1)^	0 Week	10 Weeks	*p*-Value ^(1)^	0 Week	10 Weeks	*p*-Value ^(1)^
Delta power (µV^2^/Hz)									
Frontal lobe	26.2 ± 9.2	23.9 ± 8.2	0.017 *	29.3 ± 14.2	21.1 ± 9.5	0.048 *	28.8 ± 9.4	25.4 ± 9.5	0.027 *
Central sulcus	37.9 ± 9.1	22.8 ± 7.7	0.001 **	28.4 ± 13.6	20.6 ± 9.4	0.023 *	26.6 ± 8.9	24.0 ± 9.4	0.164
Temporal lobe	24.5 ± 7.9	23.1 ± 7.0	0.256	21.2 ± 14.5	22.0 ± 9.9	0.167	26.4 ± 8.7	23.8 ± 9.5	0.125
Parietal lobe	23.2 ± 8.6	21.4 ± 8.9	0.053	27.0 ± 14.2	20.7 ± 9.2	0.106	25.7 ± 9.1	21.9 ± 8.3	0.059
Occipital lobe	21.6 ± 8.7	20.4 ± 9.1	0.451	25.0 ± 14.3	18.6 ± 8.7	0.127	25.4 ± 10.7	22.7 ± 9.9	0.153
Alpha 2 power (µV^2^/Hz)									
Frontal lobe	7.0 ± 4.4	8.4 ± 5.9	0.099	5.5 ± 2.3	6.2 ± 1.9	0.318	5.8 ± 2.4	6.1 ± 2.2	0.407
Central sulcus	6.2 ± 2.3	7.0 ± 2.5	0.019 *	5.5 ± 1.6	6.5 ± 1.7	0.081	6.2 ± 2.6	6.7 ± 2.5	0.230
Temporal lobe	6.8 ± 3.4	7.6 ± 3.5	0.043 *	57 ± 2.1	5.9 ± 2.0	0.708	6.3 ± 2.6	6.5 ± 2.5	0.653
Parietal lobe	8.1 ± 4.3	9.7 ± 6.1	0.024 *	6.3 ± 2.4	6.9 ± 2.2	0.383	7.0 ± 2.8	8.2 ± 3.4	0.005 **
Occipital lobe	8.6 ± 6.2	10.4 ± 8.0	0.076	6.9 ± 2.7	7.5 ± 2.9	0.502	7.2 ± 3.2	7.4 ± 3.1	0.605
Beta 1 power (µV^2^/Hz)									
Frontal lobe	5.1 ± 1.8	5.5 ± 1.7	0.012 *	5.4 ± 2.4	6.0 ± 2.3	0.308	4.6 ± 1.6	5.3 ± 1.4	0.007 **
Central sulcus	5.9 ± 1.7	6.6 ± 2.2	0.009 **	5.8 ± 1.9	6.5 ± 1.9	0.154	5.7 ± 2.0	6.2 ± 1.8	0.099
Temporal lobe	5.6 ± 1.7	5.9 ± 1.7	0.059	5.5 ± 2.2	5.8 ± 1.8	0.646	5.0 ± 1.7	5.4 ± 1.5	0.101
Parietal lobe	6.5 ± 2.7	7.1 ± 2.6	0.019 *	5.4 ± 2.6	7.1 ± 2.3	0.344	5.9 ± 2.1	6.5 ± 2.2	0.057
Occipital lobe	5.8 ± 2.3	6.1 ± 2.2	0.391	5.4 ± 2.9	6.0 ± 2.0	0.503	5.4 ± 2.3	5.6 ± 1.9	0.529

Values are presented as means ± standard deviations or numbers (%). ^(1)^ Significance as determined by a paired *t*-test, with a *p*-value less than *p* < 0.05. * *p* < 0.05, ** *p* < 0.01.

## Data Availability

The data presented in this study are available from the corresponding author upon reasonable request.

## Abbreviations

The following abbreviations are used in this manuscript:

AI	Artificial Intelligence
MCI	Mild Cognitive Impairment
LDL	Low-Density Lipoprotein
HDL	High-Density Lipoprotein
MNA-SF	Mini Nutritional Assessments-Short Form
K-MMSE-2 SV	Korean Version Of Mini-Mental State Examination-2 Standard Version
K-ADL	Korean Version Of The Activities Of Daily Living
EEG	Electroencephalography
LTC	Long-Term Care
BMI	Body Mass Index
BFM	Body Fat Mass
PBF	Percent Body Fat
SMM	Skeletal Muscle Mass
SBP	Systolic Blood Pressure
DBP	Diastolic Blood Pressure
